# Rationale, design and baseline characteristics of a randomized controlled trial of a web-based computer-tailored physical activity intervention for adults from Quebec City

**DOI:** 10.1186/s12889-015-2364-3

**Published:** 2015-10-09

**Authors:** François Boudreau, Michel Jean Louis Walthouwer, Hein de Vries, Gilles R. Dagenais, Ginette Turbide, Anne-Sophie Bourlaud, Michel Moreau, José Côté, Paul Poirier

**Affiliations:** Département des sciences infirmières, Université du Québec à Trois-Rivières, 3351, boul. des Forges, P.O. Box 500, Trois-Rivières, Qc G9A 5H7 Canada; Department of Health Promotion, School for Public Health and Primary Care (CAPHRI), Maastricht University, Maastricht, Netherlands; Institut Universitaire de Cardiologie et de Pneumologie de Québec, Québec, Qc Canada; Département de médecine, Faculté de médecine, Université Laval, Québec, Qc Canada; Faculté des sciences infirmières, Centre de recherche du Centre hospitalier de l’Université de Montréal, Montréal, Qc Canada; Faculté de Pharmacie, Université Laval, Québec, Qc Canada

**Keywords:** Physical activity, Adult, Computer-tailoring, Videos, Cardiovascular disease, eHealth

## Abstract

**Background:**

The relationship between physical activity and cardiovascular disease (CVD) protection is well documented. Numerous factors (e.g. patient motivation, lack of facilities, physician time constraints) can contribute to poor PA adherence. Web-based computer-tailored interventions offer an innovative way to provide tailored feedback and to empower adults to engage in regular moderate- to vigorous-intensity PA. To describe the rationale, design and content of a web-based computer-tailored PA intervention for Canadian adults enrolled in a randomized controlled trial (RCT).

**Methods/Design:**

244 men and women aged between 35 and 70 years, without CVD or physical disability, not participating in regular moderate- to vigorous-intensity PA, and familiar with and having access to a computer at home, were recruited from the Quebec City Prospective Urban and Rural Epidemiological (PURE) study centre. Participants were randomized into two study arms: 1) an experimental group receiving the intervention and 2) a waiting list control group. The fully automated web-based computer-tailored PA intervention consists of seven 10- to 15-min sessions over an 8-week period. The theoretical underpinning of the intervention is based on the I-Change Model. The aim of the intervention was to reach a total of 150 min per week of moderate- to vigorous-intensity aerobic PA.

**Discussion:**

This study will provide useful information before engaging in a large RCT to assess the long-term participation and maintenance of PA, the potential impact of regular PA on CVD risk factors and the cost-effectiveness of a web-based computer-tailored intervention.

**Trial registration:**

ISRCTN36353353 registered on 24/07/2014

## Background

In Canada, cardiovascular disease (CVD) is a major cause of mortality, morbidity and health cost [1]. Appropriate management of several risk factors contributing to CVD is poorly achieved even among patients with this disease [2]. Promoting physical activity (PA) is one of the key strategies to reduce CVD [3]. Unfortunately, recent population surveillance data indicated that 85 % of adults do not meet Canada’s PA guideline of 150 min of moderate-to-vigorous-intensity PA per week (4). This paper describes the rationale, study design, development and content of the web-based computer-tailored PA intervention as well as the baseline characteristics of adults men and women from Quebec City enrolled in this randomized trial.

### Rationale of the study

#### Using a web-based computer-tailoring intervention

Patient motivation [4], use of local area facilities for PA [5] and physician time constraints [6] may be elements contributing to the poor PA achievements among Canadian adults. Web-based computer-tailored interventions offer an interesting alternative approach in motivating people to change their PA behaviour [7]. This technology follows principles of face-to-face counselling and assesses individuals’ perceived health behaviour status as well as the determinants that influence their motivation and behaviour. Next, tailored feedback is provided about these determinants based on individuals’ answers and personal characteristics [7, 8]. According to the Elaboration Likelihood Model [9], the provision of tailored feedback will result in more thoughtful information processing via the central route of persuasion since tailored messages are perceived as being personally relevant and thus encourage the person to pursue the desired behaviour. This is suggested by functional magnetic resonance imaging studies showing that people pay more attention to tailored smoking-cessation messages because of their personal relevance [10, 11]. The benefits of web-based computer-tailored interventions include flexibility in the time and location of access for the user, no required contact with healthcare professionals and the possibility to reach many users under free-living conditions. Hence, a web-based computer-tailored intervention offers an innovative and accessible way to target people’s PA behaviour in order to promote cardiovascular health [12, 13].

Systematic reviews [14, 15] and meta-analyses [16, 17] have shown promising findings of web-based computer-tailored interventions in modifying PA behaviour. For instance, Krebs et al.’s meta-analysis of 25 studies showed a pooled mean effect size (Hedges’ *g*) of 0.16 (*p* < 0.001), and that 43 % of participants receiving a tailored intervention adhered to the PA recommendations at follow-up compared to 34 % in the control group [16]. Meta-analyses highlighted some characteristics that increase the likelihood of positive results [16, 18]. Using dynamically tailored interventions (i.e. updating feedback to reflect a person’s changes) [16], designing PA messages based on social-cognitive theories [18] and using more behaviour change techniques [18] should all be considered in developing such interventions. Moreover, recent studies have shown that a web-based computer-tailored intervention can be cost-effective in the PA domain [19] and in other health-related behaviours as well [20]. Therefore, a web-based computer-tailored intervention was developed to improve PA levels among Canadian adults particularly women and people with low-to moderate-education levels.

#### Targeting specific population

Within this intervention, particular attention was given to women and people with a low-to-moderate education level in preparation for the content before the recruitment. While Canadian women are more likely than Canadian men to engage in preventive behaviour with respect to CVD [21], PA is the only behaviour where men outperform women across all age groups [21, 22]. It has been suggested that PA is a gendered experience and the secondary cause of the differences in the observed PA rates are partially due to the role of gender in the social, economic and health aspects of women’s lives. Factors such as early parenthood and career demands [23], lack of time due to family responsibilities [24] and less social support [25] may explain differences in PA levels in women and men. In order to increase PA levels among women, this web-based computer-tailored intervention will pay special attention to beliefs and issues that are specific to women.

In Canada, people with hypertension characterized by a low (less than secondary graduation) or moderate (secondary graduation) level of education are less likely to engage in PA for blood pressure control [26], which is one of the major CVD risk factors. Hence, there is a need to focus interventions on these people in particular. As hypothesized by Walthouwer et al. [27], a video intervention might be more appealing and effective for people with a low educational level. Based on the principles of the Elaboration Likelihood Model [9] and the I-Change Model [8], it can be reasoned that the use of online video information may increase attention, comprehension and appraisal better than traditional text-based approaches among people with lower levels of education. Positive effects in people with lower levels of education are expected since they encounter greater difficulty when translating abstract text into concrete actions. Moreover, Alley et al.’s [28] findings highlighted that video-based messages were more effective at gaining participants’ visual attention than text-based messages in a web-based PA intervention.

### Methods/Design

#### Objectives and design

The aim of the study is to test the efficacy and the use of a web-based computer-tailored intervention targeting PA in adults not reaching Canada’s physical activity guidelines. The first objective is to determine the efficacy of the web-based computer-tailored intervention after 3 months and include two specific questions: 1) what is the efficacy of the intervention on PA behaviour? 2) how are the intervention effects on PA behaviour mediated by motivational factors? The second objective is to examine the use of the web-based computer-tailored intervention and include a specific question: do the intervention initiation and intervention completion differ in relation to sex and level of education?

The study is a RCT with two study arms: 1) an experimental group receiving the intervention and 2) a waiting list control group (for whom the intervention will be offered afterwards). This strategy was employed in order to increase acceptability and adherence to the research protocol for the participants in the control group, and thereby to decrease attrition effects [29]. Excel software with the function RAND was used to randomize participants in both the experimental group and the control group.

#### Participant eligibility and recruitment

Men and women aged between 35 and 70 years without CVD and physical disability or other limitations reducing their ability to walk, not participating in regular moderate- to vigorous-intensity PA, familiar with and having home access to a computer and with signed informed consent, were recruited from the Quebec City Prospective Urban and Rural Epidemiological (PURE) study centre. The PURE study is an ongoing epidemiological study including 156,000 men and women aged 35 to 70 years living in urban and rural regions of 17 countries with different economic levels in 2007 assessing the causes of chronic non-communicable diseases [30]. Participants in this cohort responded to standardized questionnaires regarding their socio-demographic and -economic characteristics, their heath profile, diet, physical activities, smoking and medications. Furthermore, they had blood pressure, weight, height, waist, hip, strength, biochemical, respiratory function and electrocardiographic measurements at entry in the study. In Canada there are 10,700 participants living in four cities; 2790 of them are from Quebec City.

In April 2014, potential participants received an email as an introduction package which contained a) an email letter from the two principal investigators (PP, GRD) of the Quebec City PURE study centre describing the purpose of the study; b) a brief information document attached to the email describing in more detail the study and an electronic participation form (one page) which participants were invited to complete and return in order to signal their interest in the study. A follow-up reminder email was sent to the non-respondents one week after emailing the introduction package.

#### Intervention development process

The intervention is based on an innovative Dutch web-based computer-tailored intervention [8, 31, 32] that has been adapted to Canadian adults, taking into account specific PA beliefs reported for Canadian adult women and men [23, 33, 34]. The most frequent beliefs were integrated into the intervention. We also conducted one additional focus group to identify the most salient beliefs associated with the practice of PA specific to women.

**Fig. 1:**
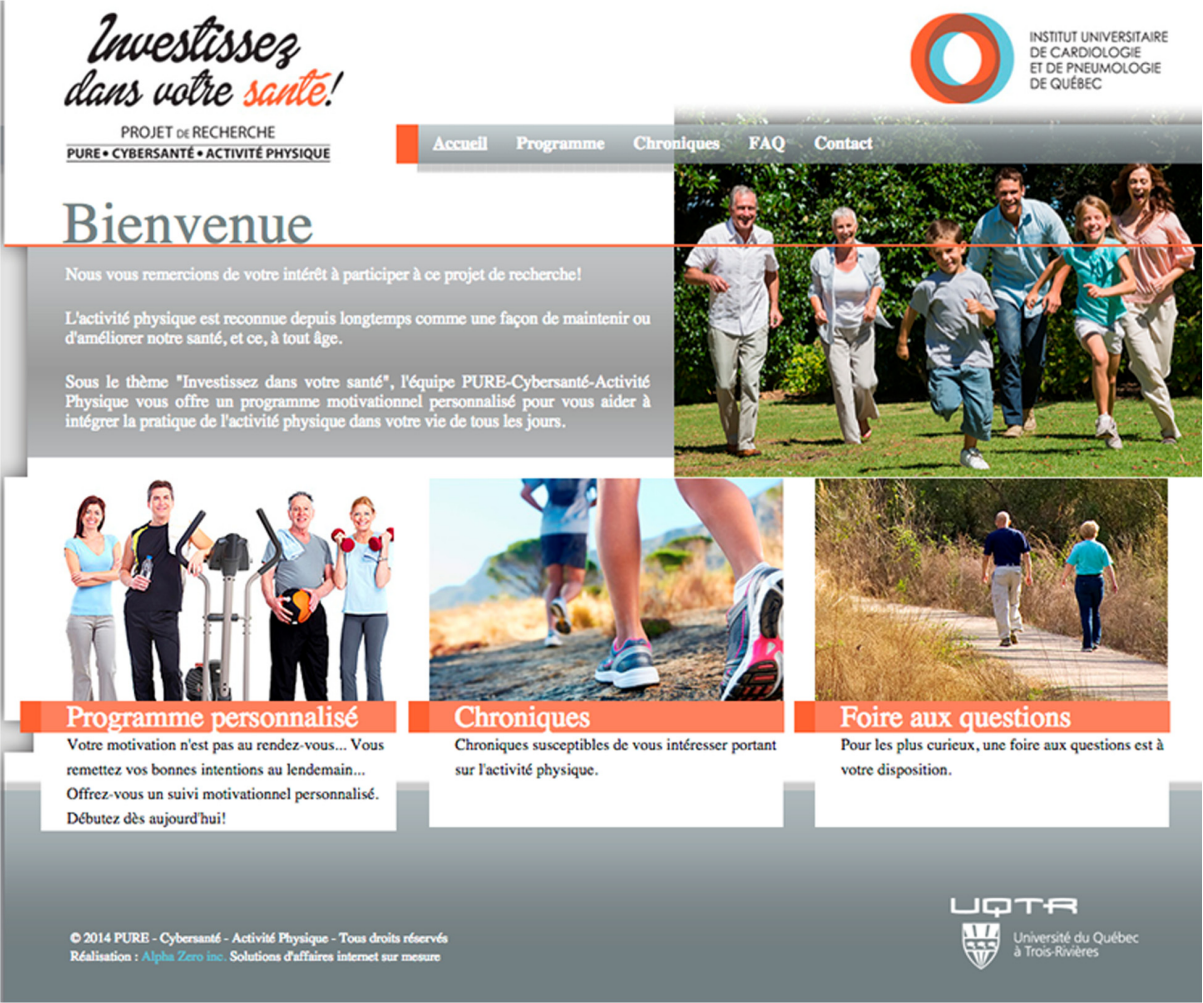
Screenshot of the PURE-eHealth-Physical Activity website

The intervention is developed using the software program Tailorbuilder (OverNite Software Europe, the Netherlands), a program specifically designed to develop computer-tailored interventions. The intervention was integrated into a website (Fig. 1) which provides study information and answers to frequently asked questions. Participants need to log in to this website to be able to interact and use the intervention. The video messages for people with a low/moderate educational level were developed by Point Bleu Productions, a professional company based in Trois-Rivières (Canada). In total, 33 videos were recorded in French in which two professional actors read the feedback messages aloud using a news-driven format. Before a final version of the intervention was developed, five people were asked to pre-test the intervention. The results of these pre-tests were used to improve the intervention and develop the final version.

**Fig. 2:**
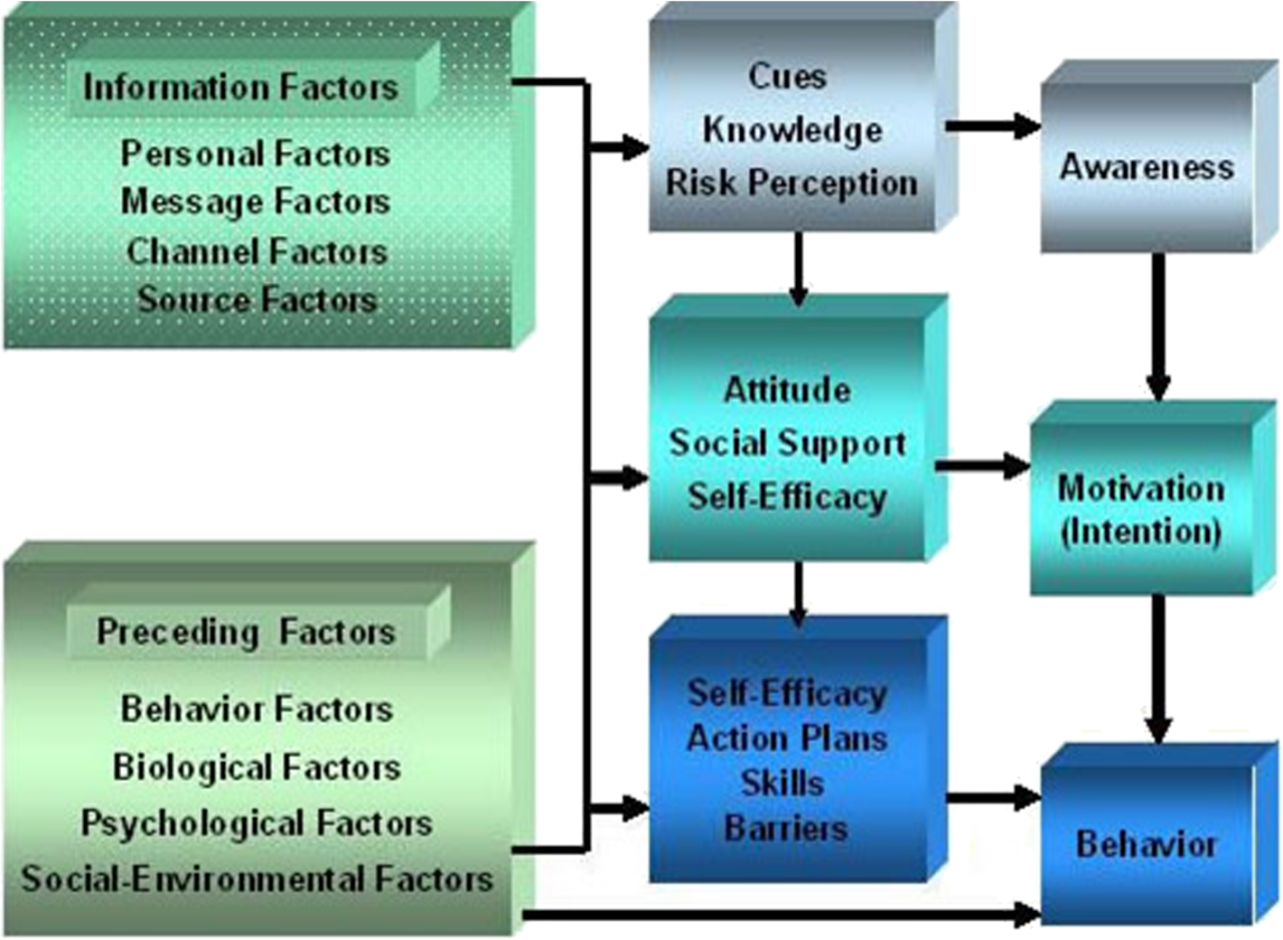
Graphical representation of the I-Change Model

The theoretical background underlying the intervention was based on the I-Change Model [8] (Fig. 2) which incorporates several socio-cognitive models, including the theory of planned behaviour [35], the Transtheoretical Model [36], the Precaution Adoption Process Model [37] and the goal-setting theory [38]. This model has been used successfully to develop computer-tailored interventions targeting various health-related behaviours. The model assumes the following three phases in the behavioural change process: (1) raising awareness about the need to change a given health behaviour; (2) motivation to change behaviour and (3) specifying actions required to translate intention into the final goal behaviour. Raising awareness assumes the existence of sufficient knowledge, the perception of high risk and the existence of internal and external cues to action. Motivation can be increased by emphasizing the advantages of the desired behaviour and tackling perceived disadvantages. An individual’s motivation can also be enhanced by increasing the perceived social support, modelling and social norms in favour of the desired behaviour and by increasing self-efficacy to perform the intended behaviour. These factors constitute a person’s level of intention towards the desired behaviour. Translation of intention into actual behaviour is dependent on a person’s skills and action planning.

#### Content of intervention

The web-based computer-tailored intervention consists of seven different sessions that each take approximately 10 to 15 min to complete. To remind participants that they could continue with the next session, an automated email was sent 7 days after completing the session; in the case of non-response, a reminder was sent 10 days after completing the last session. In the intervention, participants receive tailored feedback on their PA level and the following I-Change Model beliefs: knowledge, risk perception, attitude, social influence, self-efficacy and intention. As emphasized, women and participants with a low level of education (elementary and secondary school) receive specific attention within the intervention. For example, women will receive feedback on additional beliefs that are particularly relevant to women (e.g. not having sufficient time because of family responsibilities). In addition, since it has been hypothesized that videos are more efficient for people with a low educational level, these participants will receive the feedback regarding their PA level and their I-Change Model beliefs via video messages instead of text. These videos have exactly the same content as the text-based messages. Each session is explained in detail below, including the additional elements for women and participants with a low educational level.

##### Session one

After a brief explanation of the study, participants are asked to provide their informed consent to participate in the study. Participants who agree to participate then start with the baseline measurement, in which several demographic characteristics, PA level and various I-Change Model variables are assessed. Participants’ answers to this questionnaire will be used to provide the tailored feedback messages in the remaining six intervention sessions. In this session, participants do not receive any tailored information. At the end of this session, participants decide for themselves if they want to continue with session two immediately or wait a few days before beginning the next session. Participants who decide to wait for the next session will receive an email after 3 days to remind them that they can start the second session.

##### Session two

This is the actual start of the intervention, and participants receive tailored feedback for the first time on beliefs such as ‘By doing regular PA in my free time, I will have better heart health’. Feedback will be accompanied by the testimony of an adult showing the benefits of regular PA for heart health. Next, participants are given the option to read more information about the Canadian PA standard and the definition of moderate- and vigorous-intensity PA. Participants will subsequently receive feedback that indicates how many minutes per week they are physically active at a moderate- to vigorous-intensity level (i.e. descriptive feedback). This feedback will also include a comparison of their PA behaviour to the Canadian standard (i.e. normative feedback). In addition, participants will also receive suggestions about how they can improve their PA level; for participants with a low educational level, this part was given using video messages. The last part of the session consists of tailored feedback about the perceived advantages and disadvantages of PA. Using a decision balance, participants are informed about whether they perceive the advantages as higher, lower or equal to the disadvantages of PA. Perceived advantages will be reinforced, while perceived disadvantages will be countered. Women receive additional feedback on two pros (e.g. less social isolation and improved quality of sleep) and two cons (e.g. muscle pain and money to engage in physical activity) that are specific for them. For participants with a low educational level, tailored videos are used to deliver this feedback. Session two is concluded by a summary of the session.

##### Session three

One week after session two, this session starts with an extensive section where participants receive tailored feedback on the following I-Change Model variables: intention, self-efficacy, social influence and attitude. For participants with a low educational level, feedback is given using tailored videos. The aim of this feedback is to further increase participants’ motivation to increase their moderate- to vigorous-intensity PA level. Participants will first receive tailored feedback on their intention to increase their PA level. Next, they will receive feedback on their self-efficacy which consists of practical information about how to deal with situations in which their self-efficacy to be physically active is low. When participants have a high self-efficacy, this perception will be reinforced. Women receive additional feedback on potential barriers that are unique to them, such as feeling unsafe, time constraints due to a combination of professional and household issues, and looking after children. Tailored feedback on social influence will be given separately for social support, modelling and social norm. The social norm feedback message will explain how participants can make use of social support. Participants then receive feedback based on their social norm score. Participants who think that they should be physically active according to people in their social environment receive reinforcing feedback. Participants with a low social norm score are informed that the norm to be physically active in general is high and they will be encouraged to talk about this with other people. Participants then receive tailored feedback on modelling by emphasizing that many of the people in their social environment are physically active as well. Feedback about participants’ attitude is exactly the same as in session two, and participants are asked whether or not they want to receive this feedback again. After this section on participants’ beliefs about PA, they are asked to set a goal. Participants are informed that this goal should be both challenging and realistic. They can set a goal by selecting how many minutes per week (from 10 to 60 min) they want to increase their moderate- and vigorous-intensity PA level. After this decision, participants are asked to indicate when they want to start this goal by setting a change date (i.e. start date). This date should be set within one to two weeks after session three. The minimum period for the change date is set at one week because participants are encouraged to first make adequate preparations (e.g. joining a gym or buying sports clothes). After participants have set a change date, they are asked to indicate which preparations they will need to undergo. They can do this by selecting a maximum of three preparations from a list of ten options. These preparations are specified as plans using implementation intentions, as this strategy increases the chance of a successful translation of intention into an actual change in behaviour. Following this decision, participants are encouraged to carry out the chosen preparatory plans as quickly as possible. The session ends with a summary of participants’ goal, change date and preparatory plans.

##### Session four

This session takes place four days before the day they want to increase their moderate- and vigorous-intensity PA level. In this session, participants receive final information to help them achieve their goal. They also receive more in-depth information about the purpose of having preparatory plans and some practical tips for carrying them out. Subsequently, participants’ self-efficacy in achieving their goal will be assessed. Based on their self-efficacy level, participants are informed about how they can achieve their goal even if they encounter difficult situations. Finally, the session ends with a general message to motivate participants to start with their goal. Participants will also receive an e-mail on the chosen change date to wish them good luck and remind them that they can start with their goal.

##### Session five

This session takes place 1 week after the change date and starts with a participant PA assessment during the past week. This will be compared to participants’ baseline score and they will be informed if their PA level improved, decreased or remained stable and whether or not they have achieved their goal. Feedback also includes a graph to visualize the comparison. For participants with a low educational level, this feedback will be provided by means of a tailored video. Below the video, a graph indicating their comparison will be shown as well. Participants who improved or who achieved their goal are complimented and encouraged to maintain their progress. If the participants’ PA level decreased or remained stable, the failure will be attributed to external factors and they will be motivated to try to achieve their goal during the upcoming week. To further increase the motivation of these participants, they will be offered the possibility to read the tailored feedback on their intention, self-efficacy, social influence and attitude from session three again. The last part of this session focuses on dealing with difficult situations (i.e. barriers). Using a list of various difficult situations, participants are given the opportunity to select a maximum of three situations that they encountered or expect to encounter in the future. To help participants deal with these situations, they receive four possible coping options for each selected difficult situation. After receiving this information, participants are given the opportunity to select their own preferred coping option from a list of various possible coping options. Following this process, participants will make a coping plan that consists of the selected difficult situation and the selected coping option. At the end of the session, participants receive an overview of their coping plans, including tips for carrying them out.

##### Sessions six and seven

Both sessions are comparable to session five and take place two and four weeks after this session, respectively. Session six also includes an additional section about how to maintain long-term behavioural changes. Participants will receive information and tips to help them monitor, plan and maintain their PA progress. Session seven is identical to session six, but the focus in the wording is on maintaining progress, or in the case of a relapse, to restart the change process. At the end of session seven, participants are further asked to complete a process evaluation about the intervention. Finally, participants will be thanked for using the intervention and informed that there will be a follow-up measurement after a few weeks in the context of the corresponding intervention study.

#### Evaluation

Participants have to complete the following two questionnaires on the study website: 1) the baseline questionnaire (T0) and 2) the follow-up questionnaire 3 months after baseline (T1). The baseline questionnaire will assess the following demographics: gender, age, marital status, education, occupation, ethnicity, weight and height. Participants’ PA level will be assessed using a validated questionnaire [39]. The psychosocial determinants of the I-Change Model related to PA (intention, attitude, social influences, self-efficacy) will be assessed on a 7-point Likert scale [27]. The 3-month follow-up questionnaire consists of PA level reassessment as well as two I-Change Model variables, intention and self-efficacy. In addition, the follow-up questionnaire also consists of process evaluation questions in order to evaluate participants’ opinion of the intervention.

#### Sample size and data analysis

##### Sample size

The sample size was calculated using the G*Power software platform [40]. According to a mixed plan 2 (intervention group, control group) × 2 (baseline, 3-month follow-up), assuming a statistical power of 0.80, an alpha value of 0.05 and a moderate correlation (*r* = 0.5) between baseline and 3-month follow-up PA measures, a total sample size of 200 participants is required to detect a small effect size (*f* = .10). Statistical analysis using Statistical Package for the Social Sciences (SPSS) software (version 21) will be performed in line with the study’s objectives.

##### Efficacy of the intervention

The efficacy of the PA intervention, 3 months after baseline, will be assessed with linear mixed models. This statistical analysis is very well suited for modelling data containing repeated measures from several participants [41] and has the advantage of providing better capabilities to handle missing observations compared to more traditional approaches, such as repeated measures ANOVA [42, 43]. It is essentially a general linear model, with the addition of random effects to account for the two sources of variability, within (e.g. time 0, time 1)- and between (e.g. intervention, waiting list control group)-subject variance [41]. Analysis will be adjusted for socio-demographic characteristics and PA variables that significantly differ between study arms at baseline.

##### Mediation effect

To explore the possible underlying mechanisms of PA change, potential mediators of the PA behaviour intervention effect will be explored. Based on the I-Change model (Fig. 2), a multiple mediation analysis with condition (intervention vs. control condition) as the independent variable; intention and self-efficacy as mediator variables and PA behaviour as the dependent variable will be conducted. Preacher and Hayes’s SPSS macro for multiple mediator models will be used for testing the mediation hypothesis [44].

##### Use of the intervention

Logistic regression will be conducted with *intervention initiation* (first Website visit, 0 = no/1 = yes) as the dependent variable and demographics status will be included in the model as predictors of intervention initiation. The same independent variables will be considered in the model, with the addition of the intention to be physically active, as predictors of *intervention completion*.

### Ethics study approval

The study protocol was approved by the ethics committees of the Université du Québec à Trois-Rivières (CER-13-192-06.32) and the Institut Universitaire de Cardiologie et de Pneumologie de Québec (21002).

### Baseline characteristics

Among the 2790 Quebec City PURE participants, 1269 were eligible to participate in the present study, of which 244 actually signed up. The socio-demographic and clinical characteristics of these 244 participants are described in Table 1. Participants had a mean age of 56 years, were mostly women (67.6 %) and have completed their college/university degree (83.6 %). Half of the population were overweight or obese, 12 % had hypertension and 2 % diabetes.

**Table 1:** Characteristics of the randomized participants

Number of participants, *n*	244		
Mean age (*SD*), [range], years	56.3	(7.0)	[39–70]
Gender, *n* (%)			
female	165	(67.6)	-
male	79	(32.4)	-
Highest education level completed, *n* (%)			
high school	40	(16.4)	-
college/university	204	(83.6)	-
Smoking behaviour, *n* (%)			
current	14	(5.7)	-
never	127	(52.0)	-
former	103	(42.2)	-
Body mass index, *n* (%), kg/m^2^			
underweight (16.5-17.99)	2	(0.8)	-
normal (18–25)	112	(45.9)	-
overweight (25.01–29.99)	90	(36.9)	-
obese (30+)	40	(16.4)	-
Mean blood pressure (*SD*), [range], mm Hg			
systolic	127.0	(18.3)	[85–189]
diastolic	80.0	(11.0)	[55–117]
Mean low-density lipoprotein level (*SD*), [range], mmol/L	3.01	(0.8)	[1.21–5.57]
Mean high-density lipoprotein level (*SD*), [range], mmol/L	1.66	(0.44)	[.83–3.20]
Mean apolipoprotein B level (*SD*), [range], mmol/L	0.91	(0.24)	[.32–1.65]
Mean glycemia level (*SD*), [range], mmol/L	5.0	(0.7)	[3.7–10.2]
Participants reported risk factors, *n* (%)			
Hypertension	29	(11.9)	-
Diabetes	5	(2.0)	-
Medications, *n* (%)			
Anti-hypertensive medication	26	(10.7)	-
Hypoglycemic medication	2	(0.8)	-

## Discussion

The main objective of this paper was to describe the rationale, design, development of the content and baseline characteristics of participants enrolled in a randomized controlled trial of a web-based computer-tailored PA intervention. We hypothesize that at the 3-month follow-up, participants in the intervention condition will show higher levels of moderate- to vigorous-intensity PA compared to the control group. In line with current priorities, we focus intervention development on two groups: women and individuals with a low-to-moderate education level. The overall participation rate was 19 %, which is comparable to other web-based, computer-tailored, PA interventions (15 % [19], 15 % [45], 29 % [46]). Women constitute two-third of the 244 participants. However, only 15 % of the participants had a low-to-moderate education levels. These participation rates are not unique to our study; other web-based PA interventions also reported that participants were mostly women [28, 45, 47–49] and highly educated [19, 28, 45, 47–51].

In the context of the Quebec healthcare system and in compliance with the recommendations of the Health and Welfare Commissioner [52], the integration of the proposed technology presents a new means of offering services that include programs to promote heart health and prevent cardiovascular disease. In the Quebec healthcare system [53], as well as elsewhere [54], lack of time is often a major impeding factor for health care professionals when it comes to face-to-face counselling. Computer-tailoring technology is one of the most significant innovations developed over the last decade in the field of support services for face-to-face counselling. People are likely to benefit from access to a web-based self-management platform that can be tailored and adapted to each individual in the comfort of their own home without being restricted to the limited time of busy health care professionals. Despite the sustained growth of this type of technology in the scientific community over the past 20 years, its use is still in the early stages in the province of Quebec. The results of this study will contribute to the development of data that will provide answers to some emerging research questions in the field of computer-tailoring, such as reaching high-risk groups [55] and establishing larger effect sizes [15].

This study has several strengths. First, the use of video-driven messages in the context of web-based computer-tailored interventions is promising and innovative. In terms of methodology, the use of a randomized controlled trial is a strength because it decreases possible bias (internal validity). Finally, by reaching participants directly in their homes (no clinical office visits), the proposed web-based intervention could have a major public health impact. The study also has limitations. The outcome measure is based on self-reporting using the Godin Leisure-Time Exercise Questionnaire. Nevertheless, it has been demonstrated that the reliability and validity of this tool compared favourably to nine other self-report measurements [56]. The Pure-eHealth-Physical Activity intervention is only available on the Internet and thus excludes participants who do not have access to the Internet, which is more likely to be the case among people with a low level of education. However, the 2009 Canadian Internet Use Survey pointed out that the gap is narrowing in terms of Internet users between the lowest (76 %) and highest (92 %) quartiles of income [57]. In addition, while the participants may know how to use the Internet, they may not be familiar or willing to comply with this new intervention. Studies such as this one are crucial to assess the participation rate and the impact of these interventions.

## Conclusion

This paper provides insight into the development of a web-based computer-tailored PA intervention for Canadian adults aged 35 to 70 years of age. The results of this study will have useful information about the feasibility, effects, appreciation and use of this intervention. This insight needs to be considered before embarking on a full-scale RCT to assess the long-term benefit and cost-effectiveness of the intervention on PA.

## References

[1] Statistics Canada (2009). Leading Causes of deaths in Canada, 2009, CANSIM Table 102–0561.

[2] Yusuf S, Rangarajan S, Teo K, Islam S, Li W, Liu L (2014). Cardiovascular risk and events in 17 low-, middle-, and high-income countries. New Eng J Med.

[3] Anderson TJ, Grégoire J, Hegele RA, Couture P, Mancini GBJ, McPherson R (2013). 2012 Update of the Canadian Cardiovascular Society Guidelines for the diagnosis and treatment of dyslipidemia for the prevention of cardiovascular disease in the adult. Can J Cardiol.

[4] Korownyk C, Allan GM (2010). Motivating patients to activity. Can Fam Physician.

[5] Riva M, Gauvin L, Richard L (2007). Use of local area facilities for involvement in physical activity in Canada: insights for developing environmental and policy interventions. Health Promot Int.

[6] Hébert ET, Caughy MO, Shuval K (2012). Primary care providers’ perceptions of physical activity counselling in a clinical setting: a systematic review. Brit J Sport Med.

[7] de Vries H, Brug J (1999). Computer-tailored interventions motivating people to adopt health promoting behaviours: introduction to a new approach. Patient Educ Couns.

[8] de Vries H, Kremers SPJ, Smeets T, Brug J, Eijmael K (2008). The effectiveness of tailored feedback and action plans in an intervention addressing multiple health behaviors. Am J Health Promot.

[9] Petty RE, Cacioppo JT (1986). Communication and persuasion : central and peripheral routes to attitude change.

[10] Chua HF, Polk T, Welsh R, Liberzon I, Strecher V (2009). Neural responses to elements of a web-based smoking cessation program. Stud Health Technol Inform.

[11] Chua HF, Liberzon I, Welsh RC, Strecher VJ (2009). Neural correlates of message tailoring and self-relatedness in smoking cessation programming. Biol Psychiatry.

[12] Neubeck L, Ascanio R, Bauman A, Briffa T, Clark AM, Freedman B (2011). Planning locally relevant Internet programs for secondary prevention of cardiovascular disease. Eur J Cardiovasc Nurs.

[13] Kerr C, Murray E, Noble L, Morris R, Bottomley C, Stevenson F (2010). The potential of Web-based interventions for heart disease self-management: a mixed methods investigation. J Med Internet Res.

[14] Neville LM, O’Hara B, Milat A (2009). Computer-tailored physical activity behavior change interventions targeting adults: a systematic review. Int J Behav Nutr Phys Act.

[15] Broekhuizen K, Kroeze W, van Poppel MNM, Oenema A, Brug J (2012). A systematic review of randomized controlled trials on the effectiveness of computer-tailored physical activity and dietary behavior promotion programs: an update. Ann Behav Med.

[16] Krebs P, Prochaska JO, Rossi JS (2010). A meta-analysis of computer-tailored interventions for health behavior change. Prev Med.

[17] Lustria MLA, Noar SM, Cortese J, Van Stee SK, Glueckauf RL, Lee J (2013). A meta-analysis of web-delivered tailored health behavior change interventions. J Health Commun.

[18] Webb TL, Joseph J, Yardley L, Michie S (2010). Using the internet to promote health behavior change: a systematic review and meta-analysis of the impact of theoretical basis, use of behavior change techniques, and mode of delivery on efficacy. J Med Internet Res.

[19] Peels DA, Hoogenveen RR, Feenstra TL, Golsteijn RH, Bolman C, Mudde AN (2014). Long-term health outcomes and cost-effectiveness of a computer-tailored physical activity intervention among people aged over fifty: modelling the results of a randomized controlled trial. BMC Public Health.

[20] Schulz DN, Smit ES, Stanczyk NE, Kremers SPJ, de Vries H, Evers SMAA (2014). Economic evaluation of a web-based tailored lifestyle intervention for adults: findings regarding cost-effectiveness and cost-utility from a randomized controlled trial. J Med Internet Res.

[21] Statistics Canada (2010). Physical activity during leisure time, 2009 (Catalogue 82–625).

[22] Colley RC, Garriguet D, Janssen I, Craig CL, Clarke J, Tremblay MS (2011). Physical activity of Canadian adults: accelerometer results from the 2007 to 2009 Canadian Health Measures Survey. sur la santéHealth Rep.

[23] Rhodes RE, Blanchard CM, Blacklock RE (2008). Do physical activity beliefs differ by age and gender?. J Sport Exerc Psychol.

[24] Brownson RC, Eyler AA, King AC, Brown DR, Shyu YL, Sallis JF (2000). Patterns and correlates of physical activity among US women 40 years and older. Am J Public Health.

[25] Hankonen N, Absetz P, Ghisletta P, Renner B, Uutela A (2010). Gender differences in social cognitive determinants of exercise adoption. Psychol Health.

[26] Gee ME, Bienek A, Campbell NRC, Bancej CM, Robitaille C, Kaczorowski J (2012). Prevalence of, and barriers to, preventive lifestyle behaviors in hypertension (from a national survey of Canadians with hypertension). Am J Cardiol.

[27] Walthouwer MJL, Oenema A, Soetens K, Lechner L, De Vries H (2013). Systematic development of a text-driven and a video-driven web-based computer-tailored obesity prevention intervention. BMC Public Health.

[28] Alley S, Jennings C, Persaud N, Plotnikoff RC, Horsley M, Vandelanotte C (2014). Do personally tailored videos in a web-based physical activity intervention lead to higher attention and recall? - an eye-tracking study. Front Public Health.

[29] Darker CD, Frenchb DP, Eves FF, Sniehotta FF (2010). An intervention to promote walking amongst the general population based on an ‘extended’ theory of planned behaviour: A waiting list randomised controlled trial. Psychol Health.

[30] Teo K, Chow CK, Vaz M, Rangarajan S, Yusuf S (2009). The Prospective Urban Rural Epidemiology (PURE) study: examining the impact of societal influences on chronic noncommunicable diseases in low-, middle-, and high-income countries. Am Heart J.

[31] Smeets T, Brug J, de Vries H (2008). Effects of tailoring health messages on physical activity. Health Educ Res.

[32] Oenema A, Brug J, Dijkstra A, de Weerdt I, de Vries H (2008). Efficacy and use of an internet-delivered computer-tailored lifestyle intervention, targeting saturated fat intake, physical activity and smoking cessation: a randomized controlled trial. Ann Behav Med.

[33] Rhodes RE, Blanchard CM, Courneya KS, Plotnikoff RC (2009). Identifying Belief-Based Targets for the Promotion of Leisure-Time Walking. Health Educ Behav.

[34] Vallance JK, Murray TC, Johnson ST, Elavsky S (2011). Understanding physical activity intentions and behavior in postmenopausal women: an application of the theory of planned behavior. Int J Behav Med.

[35] Ajzen I (1991). The theory of planned behavior. Organ Behav Hum Decis Process.

[36] Prochaska JO, Velicer WF (1997). The transtheoretical model of health behavior change. Am J Health Promot.

[37] Weinstein N, Lyon J, Sandman P, Cuite C (1998). Experimental Evidence for Stages of Health Behavior Change: The Precaution Adoption Process Model Applied to Home Radon Testing. Health Psychol.

[38] Locke EA, Latham GP, O’Neil HF, Drillings M (1994). Goal setting theory. Motivation: Theory and research.

[39] Godin G, Shephard RJ. A simple method to assess exercise behavior in the community. Can J Appl Sport Sci. 1986;141–6. 4053261

[40] Erdfelder E, Faul F, Buchner A (1996). GPOWER: A general power analysis program. Behav Res Methods Instrum Comput.

[41] Hayat MJ, Hedlin H (2012). Modern statistical modeling approaches for analyzing repeated-measures data. Nurs Res.

[42] Gueorguieva R, Krystal JH (2004). Move over anova: Progress in analyzing repeated-measures data andits reflection in papers published in the archives of general psychiatry. Arch Gen Psychiatry.

[43] Krueger C, Tian L (2004). A comparison of the general linear mixed model and repeated measures ANOVA using a dataset with multiple missing data points. Biol Res Nurs.

[44] Preacher KJ, Hayes AF (2008). Asymptotic and resampling strategies for assessing and comparing indirect effects in multiple mediator models. Behav Res Methods.

[45] Compernolle S, Vandelanotte C, Cardon G, De Bourdeaudhuij I, De Cocker K (2015). Effectiveness of a web-based, computer-tailored, pedometer-based physical activity intervention for adults: A cluster randomized controlled trial. J Med Internet Res.

[46] van den Brekel-Dijkstra K, Rengers AH, Niessen MA, de Wit NJ, Kraaijenhagen RA. Personalized prevention approach with use of a web-based cardiovascular risk assessment with tailored lifestyle follow-up in primary care practice - a pilot study. Eur J Prev Cardiol. 2015. 10.1177/2047487315591441 26080811

[47] De Cocker K, Spittaels H, Cardon G, De Bourdeaudhuij I, Vandelanotte C (2012). Web-based, computer-tailored, pedometer-based physical activity advice: development, dissemination through general practice, acceptability, and preliminary efficacy in a randomized controlled trial. J Med Internet Res.

[48] Vandelanotte C, De Bourdeaudhuij I, Sallis JF, Spittaels H, Brug J (2005). Efficacy of sequential or simultaneous interactive computer-tailored interventions for increasing physical activity and decreasing fat intake. Ann Behav Med.

[49] Soetens KCM, Vandelanotte C, de Vries H, Mummery KW. Using online computer tailoring to promote physical activity: A randomized trial of text, video, and combined intervention delivery modes. J Health Commun. 2014;1–16. 10.1080/10810730.2014.894597 24749983

[50] Vandelanotte C, De Bourdeaudhuij I (2003). Acceptability and feasibility of a computer-tailored physical activity intervention using stages of change: project FAITH. Health Educ Res.

[51] Spittaels H, De Bourdeaudhuij I, Brug J, Vandelanotte C (2007). Effectiveness of an online computer-tailored physical activity intervention in a real-life setting. Health Edu Res.

[52] Commissaire à la santé et au bien-être (2010). Rapport d’appréciation de la performance du système de santé et de services sociaux 2010 : adopter une approche intégrée de prévention et de gestion des maladies chroniques (recommandations, enjeux et implications).

[53] Soto JC, Chauvet ML, Groulx S, Provost S (2010). The practice and acceptance of physician preventive medicine services in a Montreal university hospital and the obstacles that deter their implementation. Can J Public Health.

[54] Yarnall KSH, Pollak KI, Østbye T, Krause KM, Michener JL (2003). Primary care: is there enough time for prevention?. Am J Public Health.

[55] Kohl LFM, Crutzen R, de Vries NK (2013). Online prevention aimed at lifestyle behaviors: a systematic review of reviews. J Med Internet Res.

[56] Courneya KS, Segal RJ, Gelmon K, Reid RD, Mackey JR, Friedenreich CM (2007). Six-month follow-up of patient-rated outcomes in a randomized controlled trial of exercise training during breast cancer chemotherapy. Cancer Epidemiol Biomarkers Prev.

[57] Statistics Canada (2010). Table 358–0126 - Canadian Internet use survey, Internet use, by location of access and income quartile.

